# Anthelmintic resistance in soil-transmitted helminths: One-Health considerations

**DOI:** 10.1007/s00436-023-08088-8

**Published:** 2023-12-20

**Authors:** Annette Imali Ng’etich, Isaac Dennis Amoah, Faizal Bux, Sheena Kumari

**Affiliations:** 1 Institute for Water and Wastewater Technology, Durban University of Technology (DUT), Durban, South Africa; 2 Department of Environmental Science, University of Arizona, Tucson, AZ USA

**Keywords:** Soil-transmitted helminths, Zoonoses, Anthelmintics, Anthelmintic resistance, One-Health

## Abstract

The One-Health approach recognizes the intricate connection between human, animal, and environmental health, and that cooperative effort from various professionals provides comprehensive awareness and potential solutions for issues relating to the health of people, animals, and the environment. This approach has increasingly gained appeal as the standard strategy for tackling emerging infectious diseases, most of which are zoonoses. Treatment with anthelmintics (AHs) without a doubt minimizes the severe consequences of soil-transmitted helminths (STHs); however, evidence of anthelmintic resistance (AR) development to different helminths of practically every animal species and the distinct groups of AHs is overwhelming globally. In this regard, the correlation between the application of anthelmintic drugs in both human and animal populations and the consequent development of anthelmintic resistance in STHs within the context of a One-Health framework is explored. This review provides an overview of the major human and animal STHs, treatment of the STHs, AR development and drug-related factors contributing towards AR, One-Health and STHs, and an outline of some One-Health strategies that may be used in combating AR.

## Background

Infections with parasitic helminths, notably soil-transmitted helminths (STHs) in humans and animals, considerably impact health and impose an immense economic burden, particularly on vulnerable communities with limited resources. These illnesses have a major influence on the food-handling chain all around the world. The World Health Organization (WHO) recognizes *Ascaris lumbricoides*, *Trichuris trichiura*, and the hookworms *Ancylostoma duodenale* and *Necator americanus* as the most prevalent human STH species (WHO 2023a). Although *A. duodenale* and *N. americanus* are frequently associated with human hookworm infections, polymerase chain reaction (PCR) analysis has detected the presence of *N. americanus* DNA in dog and pig fecal samples, implying that dogs and pigs play a likely role in the transmission of *N. americanus* in endemic regions (Boyko et al. 2020). In this review, the term STHs is broadened to include canine STHs (cSTHs) such as the hookworms *Ancylostoma caninum*, *Ancylostoma ceylanicum*, *Uncinaria stenocephala*, and *Ancylostoma braziliense*, which are zoonotic (Massetti et al. 2022) and are capable of causing cutaneous larva migrans (CLM) in humans (Traub et al. 2021). Patent *A. ceylanicum* infections manifest with accompanying clinical signs such as diarrhea and anemia (Colella et al. 2021; Stracke et al. 2020; Traub et al. 2021). The canine nematode *Toxocara canis*, which causes severe human disease, is a parasite of concern in young children in contact with pet animals and/or contaminated soil (Yousefi et al. 2020). *T. canis* larvae migrating into the bloodstream can induce a variety of clinical symptoms such as visceral larva migrans (VLM), ocular larva migrans (OLM), covert toxocariasis (CT), and neurological toxocariasis (NT) (Schwartz et al. 2022; WHO 2019b; Yousefi et al. 2020). Similarly, *Baylisascaris procyonis*, the raccoon roundworm, is a rare but serious cause of neurologic and ocular diseases in humans (CDC 2019a).

Poor environmental sanitation, insufficient safe water supply, and low socioeconomic standing, which are common characteristics of populations in low- and middle-income countries (Ihnacik et al. 2022; Montresor et al. 2020; Tinkler 2020), promote the high prevalence indexes in these settings. Furthermore, the wet and warm soils of the world’s tropical and subtropical regions promote the growth of parasite stages, which can directly infect exposed humans or animals (Ihnacik et al. 2022). While mortality rates for these parasitic infections are low, the range of health consequences and disabilities (Goshu et al. 2021) in vulnerable groups such as pre-school aged children (PSAC), school-aged children (SAC), and women of reproductive age (WRA) (Zeynudin et al. 2022), as well as young animals (Baiak et al. 2019; Kelleher et al. 2020), calls for significant efforts towards their control.

For decades, highly effective broad-spectrum anthelmintic (AH) medications with wide safety margins have been the major option for combating and controlling gastrointestinal illnesses (Baiak et al. 2019; Beleckė et al. 2021) in people and animals (livestock and companion animals) (Kotze et al. 2020). The livestock industry has greatly relied on these medicines despite the availability of several other alternative methods of parasite control such as genetic selection and pasture management. AH drugs are divided into groups based on their identical chemical structure and mode of action (Mphahlele et al. 2019a). The benzimidazoles (BZs), albendazole (400 mg), and mebendazole (500 mg) are the recommended AHs for treatment and control of human STHs in mass drug administration (MDA) campaigns (W.H.O. 2017; WHO 2023a). Hosts of different species can be treated with the same drug classes due to their similar chemical composition and mechanisms of action. For instance, BZs, such as albendazole (ALB), are widely administered across several host species, including most livestock, humans, and companion animals; macrocyclic lactones (MLs) (avermectins, e.g., ivermectin, and milbemycins, e.g., moxidectin) are used for the treatment of ectoparasite, endoparasites of livestock and companion animals, onchocerciasis, lymphatic filariasis, and *Strongyloides* in humans; pyrimidines (PYRs) (e.g., pyrantel) are occasionally administered to humans, but common in companion animals; levamisole (LEV) (an imidazothiazole) is used for gastrointestinal nematode (GIN) control in livestock (Kotze et al. 2020; Zajac and Garza 2020). Regular treatment with AHs enhanced animal productivity, vitality, and weight gain (Gilleard et al. 2021; Kelleher et al. 2020) and reduced morbidities in humans, thus justifying regular treatments (Fissiha and Kinde 2021). The low infection intensities in hosts, in turn, lead to minimal contamination of the environment with infective eggs/larvae, thus reducing reinfection after treatment. Despite these benefits, substantial rates of GINs and STH infections continue to be documented in veterinary and clinical settings.

Many years of intensive and incorrect use of AHs, however, have culminated in the rapid development of anthelmintic resistance (AR) (Beleckė et al. 2021; Fairweather et al. 2020; Kelleher et al. 2020; Kotze et al. 2020; Vineer 2020). Parasite populations frequently exposed to AHs gradually evolved from fully susceptible to fully resistant to different drugs and at different speeds. Compared with the development of antibiotic resistance in bacteria, resistance to anthelmintic drugs in nematodes has been slower to develop under field conditions (MSD Veterinary Manual 2022). The β-tubulin isotype 1 gene mutations at positions 167, 198, and 200 cause genetic resistance to BZ drugs in veterinary nematodes, thus lowering their susceptibility to treatment (Erez and Kozan 2018; Haftu et al. 2020). Small ruminants (Beleckė et al. 2021; Claerebout et al. 2020; Hinney et al. 2020; Potârniche et al. 2021) and horses (Nielsen et al. 2020; Reinemeyer 2012) are the most affected livestock hosts. It is worth noting that there is rising concern regarding the emergence of multi-drug resistance to BZs and MLs in canine hookworms (*A. caninum*) mainly in the United States of America (USA) (Castro et al. 2021; Jimenez Castro et al. 2019; Kitchen et al. 2019). Elsewhere, in Canada, the widespread use of antiparasitic drugs without the assessment of efficacy increased *A. caninum* prevalence in various Canadian provinces, and the importation of dogs, mostly from the USA, with a history of persistent *A. caninum* infections is considered a possible factor that may lead to the development of resistant isolates (Nezami et al. 2023). Resistance in other AH groups is only partially understood. Mutations in ligand-gated chloride channels and protein transporters, notably P-glycoproteins (Pgps), have been linked to ML resistance in livestock, as highlighted in a review by Verma et al. (2018). In human STHs, the presence of similar SNPs might be the cause for potential resistance emergence in *T. trichiura* (Diawara et al. 2013) and *N. americanus* (Orr et al. 2019; Rashwan et al. 2016). More concerns regarding resistance in clinical settings are compounded by reports of decreasing response to treatment with BZs (Farias et al. 2023; Vlaminck et al. 2019; Walker et al. 2021). Furthermore, the resistance patterns in veterinary species provide compelling evidence of the possibility of widespread resistance in related human species, especially with the current community-wide MDA programs. Although there is no conclusive evidence of AH resistance in human STH species, many questions still linger as to whether there is any link between the isotype-1 β-tubulin SNPs and the possible emergence of BZ resistance in human STHs (Furtado et al. 2019; Grau-Pujol et al. 2022; Matamoros et al. 2019; Orr et al. 2019; Zuccherato et al. 2018). However, since some of the parasites are zoonotic and capable of producing patent infections in people, the prospect of AR developing in clinical settings cannot be disregarded any longer.

One-Health is an integrated, unifying approach that aims to sustainably balance and optimize the health of people, animals, and ecosystems (OHHLEP 2021). This approach recognizes that the health of humans, domestic and wild animals, plants, and the wider environment (including ecosystems) is closely linked and interdependent (OIE 2023). This is because the health of animals and the environment strongly depend on human activities and human relationship with nature. Similarly, the health of animals and the environment also determine human health (OIE 2023). Therefore, embracing partnerships and engaging with experts from multiple sectors contribute to protecting health, while also addressing other health issues of concern, including antimicrobial resistance (AMR), communicable and non-communicable diseases, shortages of fresh potable water, pollution, environmental contaminants, and climate change (OHHLEP 2021). STHs are a One-Health issue because their transmission is highly dependent on contaminated environments (soils, through irrigation with untreated sewage) with infective eggs/larvae, reviewed by Amoah et al. (2018), and the presence of vertebrate animals near human dwellings that serve as reservoir hosts and influence the risk of zoonotic STH transmission. Similarly, resistance to AHs can rapidly spread among humans, animals, and the environment, owing to the excessive use of AHs in agriculture, livestock husbandry, and human medicine, contaminating plants, fresh waterways, and soils, thus underlining the relevance of One-Health in tackling this global health problem. In this review, we have extensively discussed the use of AHs in veterinary parasites and the possible connection with the emergence of resistance in human-related STH species. We have highlighted the similarity in AHs used and the occurrence of resistance-associated SNPs in both settings. Finally, we have proposed integrated approaches to minimize the use of AHs, thus protecting current and future AHs, as well as human, animal, and environmental health.

## Method of literature search

A review of peer-reviewed literature for topics on STHs, OR zoonotic infections, OR drug resistance, OR One-Health, was conducted through a search of PubMed, Google Scholar, and Science Direct. Table 1 shows additional search phrases that were utilized.

**Table 1:** The search terms used in literature search

Domain	Search term
Drug resistance	Anthelmintic resistance, gastrointestinal nematodes, OR parasitic resistance, OR nematode resistance, AND *A. lumbricoides*, OR *Ascaris suum*, OR *A. caninum*, OR *A. duodenale*, OR *T. trichiura*, OR *Trichuris suis*, AND small ruminants, OR farmed ruminants, OR grazing ruminants, OR horses, OR cattle
Zoonoses	Zoonosis, OR zoonotic infections, OR cross infections, OR zoonotic cSTHs, AND intestinal nematodes, OR intestinal parasites, OR intestinal helminths, AND *A. lumbricoides*, OR *A. suum*, OR *A. caninum*, OR *A. braziliense*, OR *T. canis*, OR *A. duodenale*, OR *T. trichiura*, OR *T. suis*, OR *A. ceylanicum*, OR *U. stenocephala*
Soil-transmitted helminths	Intestinal helminthes, OR gastrointestinal helminths, OR intestinal parasites, OR cSTHs, OR geo-helminths, AND *A. lumbricoides*, OR *A. suum*, OR *A. caninum*, OR *A. duodenale*, OR *A. caninum*, OR *A. ceylanicum*, OR *A. braziliense*, OR *T. trichiura*, OR *T. suis*, OR *T. canis*, OR *U. stenocephala*
One-Health	One-Health, AND One-Health approach, OR One-Health concept, OR One-Health triad, AND zoonotic STHs

To ensure that all published articles about the topic were included in the search results, every query was limited to the English language without any limitations on the date of publication. The bibliographies of the articles found through the search were checked for any other articles relevant to the topic. The search was expanded to include articles from organizational publications and “grey” literature, such as the World Bank, Centers for Disease Control and Prevention, World Health Organization, and European Centre for Disease Prevention and Control, the British Veterinary Association, and veterinary manuals.

## The epidemiology of STHs

The most prevalent parasitic helminths found in animal and human guts are nematodes (roundworms), cestodes (tapeworms), and trematodes (flatworms) (Salam et al. 2021). Intestinal helminth parasites of humans, also known as geohelminths or STHs, are divided into four species: *A. lumbricoides* (roundworm), *T. trichiura* (whipworm), *A. duodenale*, and *N. americanus* (Hookworms) (Goshu et al. 2021; Salam et al. 2021; WHO 2023a; Wit et al. 2021). Normally, these parasites are addressed as a group because they need similar diagnostic procedures and respond to the same medicines. An estimated 1.5 billion people, approximately 24% of the world’s population, are infected (WHO 2023a), usually with one or several species (Ahiadorme and Morhe 2020). The majority of the infections occur in tropical and subtropical regions of the world, with Sub-Saharan Africa, the Americas, China, and East Asia recording the highest prevalences (Leta et al. 2020; WHO 2023a).

Warm temperatures and adequately moist soils promote the survival, multiplication, and propagation of these parasites, whereas poverty, inadequate sanitation, and hygiene (Allan et al. 2020; Leta et al. 2020; Mpaka-Mbatha et al. 2023) are major risk factors for infection. Similarly, because of its subtropical weather, high prevalences of helminth infections among vulnerable communities have been recorded in KwaZulu-Natal, South Africa (Kjetland et al. 2020; Mpaka-Mbatha et al. 2023; Sacolo-Gwebu et al. 2019). Worm eggs present in the feces of infected humans contaminate soils in which they mature to become infective and are transmitted via ingestion of the embryonated eggs (*Ascaris* and *Trichuris* spp.) or skin penetration by the hatched L3 larvae (hookworm spp.). In addition to percutaneous infection, *A. duodenale* can be transmitted through the ingestion of larvae (CDC 2020). Incidences of human infections with the zoonotic hookworm *A. ceylanicum* are becoming more common among residents or travelers to the Asia-Pacific region (Colella et al. 2021), with more reports of autochthonous transmissions in South America and Europe (Del Giudice et al. 2019; Sears et al. 2022). Although rare, the canid *Trichuris vulpis* and pig *Trichuris suis* are the only ones that can cause persistent active infections in humans (Dunn et al. 2002; Márquez-Navarro et al. 2012), with the possibility for cross-infections of *T. suis* in humans occurring in sympatric settings (Nissen et al. 2012). *B. procyonis* is widespread among raccoons in the USA (California, Washington, Minnesota, New York) and Canada, as well as in humans in these regions, although trade of live raccoons has led to its introduction in many parts of Europe, China, and Japan (CDC 2019a).

### Burden and impact of STH infections

Given that fecal contamination of soil is essential for STH life cycles, the burden of infection is higher in underdeveloped nations where sanitary facilities are sparse and hygiene practices are poor (Mekonnen et al. 2020). The burden of STHs is projected to be 3.3 million disability-adjusted life years (DALYs) (WHO 2023a), which is a measure of years of life lost from premature death (YLL) and years of life lived with disability (YLD) of specified severity and duration (WHO 2023b). Morbidity and the burden of the disease resulting from STH infection are directly related to the intensity of the infection and its chronic nature (Zeynudin et al. 2022). As a consequence, moderate and heavy infection intensity, in addition to chronic STH infection, can cause and contribute to anemia, malnutrition, growth stunting, low birth weight, physical and cognitive impairment, and decreased school performance, and so have a detrimental impact on economic development (Blouin et al. 2018; Goshu et al. 2021; Pabalan et al. 2018). PSACs, SACs, and WRA (WHO 2023a; Zeynudin et al. 2022) are more prone to infections and suffer adverse health effects (Djuardi et al. 2021; Pabalan et al. 2018; WHO 2023a). In puppies, traumatic lesions to the intestinal mucosa caused by hematophagous hookworm parasites can result in anemia, which might prove fatal if not treated promptly (Raza et al. 2018). In animal husbandry, a heavy infestation of parasitic GINs in calves affects growth, causes gastrointestinal disorders, and may result in death (Baiak et al. 2019; Kelleher et al. 2020). On the contrary, light-intensity infections are usually asymptomatic. The common clinical manifestation is CLM or “creeping eruptions” (CDC 2019b), described as a linear or serpiginous cutaneous track that is slightly elevated, erythematous, and mobile, usually self-limiting, caused by migration of the immature larvae of mainly *Ancylostoma braziliense*, and occasionally, *Ancylostoma caninum* or *Uncinaria stenocephala* in the patient’s epidermis (CDC 2019b; Daba et al. 2021; Del Giudice et al. 2019).

## Treatment of STH infections

Infections with STHs are accompanied by various clinical complications and sequelae in humans as well as animals, resulting in significant morbidities and economic losses. The inability to break these parasites’ life cycles has been hampered by a lack of effective vaccines and inadequate sanitation and hygiene facilities, particularly in poor endemic areas, where these infections are prevalent. Accordingly, to reduce the morbidity and mortality associated with these infections, the continuous and routine use of chemical treatment with AHs (pharmacotherapy) becomes necessary. The use of AHs has been a major strategy for decades to control these infections in humans and animals (Kalkal et al. 2020; Peña-Espinoza 2018; Sangster et al. 2018; WHO 2023a; Zajíčková et al. 2020). Anthelmintic chemicals are divided into groups based on their identical chemical structure and mechanisms of action (Mphahlele et al. 2019a), but they ultimately exhibit selective toxicity to parasites (Bereda 2022) (Table 2). These mechanisms of action allow AHs to selectively target and eliminate parasites while minimizing harm to the host. By specifically targeting the parasite’s essential metabolic processes or structures, AHs disrupt their ability to survive and reproduce, ultimately leading to their eradication. Additionally, the pharmacokinetic properties of AHs enable them to accumulate in higher concentrations within parasite cells, further enhancing their efficacy against the parasites (MSD Veterinary Manual 2022).

**Table 2:** Anthelmintic drug classes, modes of action, and the mechanism of resistance

Drug class	Mode of action	Mechanism of resistance	References
Benzimidazoles	Inhibiting polymerization of microtubules	Altered target structure (β-tubulin isotype 1 mutation), β-tubulin isotype 2 mutations, deletion, altered metabolism and/or uptake	(Bereda 2022; Fourriere et al. 2020; MSD Veterinary Manual 2022; Erez and Kozan 2018)
Macrocyclic lactones (MLs)	Allosteric modulators of the glutamate-gated chloride channels (GluCls)	Mutations in GluCl and/or GABA-R genes, overexpression of P-glycoproteins, altered target (structure of GluCl channel & subunits)	(Bereda 2022; Martin et al. 2021; Moreno et al. 2021; MSD Veterinary Manual 2022)
Tetrahydropyrimidines-imidazothiazoles	Agonists of the nicotinic acetylcholine receptor (nAChR)	Changes in nicotinic acetylcholine receptors	(Bereda 2022; MSD Veterinary Manual 2022)
Aminoacetonitrile derivatives (AADs)	Agonists, allosteric modulators of the MPTL-1 channel belonging to nicotinic acetylcholine receptor	Unknown	(Bereda 2022; MSD Veterinary Manual 2022)
Spiroindoles	An antagonist of B-subtype, nAChR; inhibits 45-pS channels	Unknown	(Bereda 2022; MSD Veterinary Manual 2022; Ruiz-Lancheros et al. 2011)
Salicylanilides	Inhibits energy metabolism by uncoupling oxidative phosphorylation	Unknown	(Zajac and Garza 2020)

Until recently, there are three main broad-spectrum AH groups in the market for veterinary animals: the BZs (e.g., fenbendazole, albendazole), the MLs (e.g., ivermectin, moxidectin, eprinomectin), and the tetrahydropyrimidines-imidazothiazoles (e.g., levamisole) (Bereda 2022; Kelleher et al. 2020; Zajac and Garza 2020). Monepantel, a member of the aminoacetonitrile derivatives (AADs), is classified as the fourth group. Derquantel which belongs to the spiroindole group is the fifth AH group and is found in the market as a combination with abamectin. Closantel, a member of the salicylanilide compounds, is a narrow-spectrum AH in group 6 (Zajac and Garza 2020). Most of these AHs are applied in veterinary animals mainly due to their efficacy against a variety of intestinal parasitic nematodes as well as ectoparasites, and a few, majorly the BZs, have been optimized for human STHs. The development of new drugs is limited by high costs and modest global markets for antiparasitic drugs and chemicals (Abongwa et al. 2017).

In 2001, the World Health Assembly unanimously endorsed a resolution (WHA54.19) urging endemic countries to start seriously tackling worm-related morbidities, including those by STHs, through periodic treatment by MDA to at-risk groups in endemic areas (WHO 2023a). This is in addition to health and hygiene education to reduce transmission and reinfection by encouraging healthy behaviors, provision of adequate sanitation facilities, and access to clean water. MDA has had a significant influence on morbidity reduction; nevertheless, the efficacy of AHs varies significantly among individual STH species, and re-infection occurs promptly following treatment, necessitating regular deworming. Thus, access to the usage of clean potable water and basic sanitation and hygiene are regarded to be critical for morbidity control (Okoyo et al. 2021). AH drugs not only benefit humans and animals by reducing infection intensities and morbidities, but they also play a crucial role in preventing the transmission of zoonotic STH infections. By decreasing soil contamination with infective eggs/larvae, these drugs effectively minimize the risk of humans contracting these infections, particularly in areas where veterinary services are limited and animals have unrestricted movement. This highlights the significant impact of AH drugs on human-animal-environment health and emphasizes their importance in STH control and prevention, especially with the current slow pace in the development of new AHs.

In the absence of vaccines for most STH species, their control will continue to rely on AH drugs. As a consequence, vigilance in detecting drug resistance in parasite populations and the efficacy of current AHs is required.

## Resistance to AHs

The introduction of AH medications enabled levels of livestock parasite control previously unattainable, resulting in considerable gains to animal health and production (Gilleard et al. 2021; Kelleher et al. 2020). The drug’s remarkable effectiveness, general good safety margin, broad-spectrum nature, and affordable price led to its widespread adoption and utilization. AR is an inheritable trait that is selected for when susceptible nematode parasite species survive treatment, reproduce, and pass resistance genes to their subsequent offspring (Mphahlele et al. 2019a). The resistance genes are initially uncommon in parasite populations or may occur as a result of mutations, but as selection progresses, their fraction in the population increases, as does the number of resistant parasites (Kotze et al. 2020). Most notably, for the BZs and MLs, extensive AR has been recorded (Gilleard et al. 2021; Mickiewicz et al. 2021; Potârniche et al. 2021; Vadlejch et al. 2021) in cattle, sheep, goat, and equine gastrointestinal nematodes. Over the years, there has been an upsurge in the prevalence of AR of varying degrees across the majority of livestock AH classes globally, posing a challenge to livestock farming’s sustainability (Haftu et al. 2020; Kotze and Prichard 2016; Mphahlele et al. 2019a; Potârniche et al. 2021; Vadlejch et al. 2021).

Since AHs within each drug class have the same mechanism of action, resistance to one AH in a given drug class is likely to be accompanied by resistance to others, of that same class (side resistance) (Abongwa et al. 2017). For instance, Fissiha and Kinde (2021) observed that resistance among BZs is considered an example of side resistance. There is also the likelihood of the development of cross-resistance from AHs of one drug class to those of another if the two drug classes share similar targets (Abongwa et al. 2017).

Genetic studies in livestock parasites have linked BZ resistance to SNPs in the β-tubulin isotype 1 gene, phenylalanine-to-tyrosine substitution at positions 200 (F200Y) and 167 (F176Y), and glutamic acid-to-alanine substitution at position 198 (G198Y/E198A) (Aboelhadid et al. 2021; Atanásio-Nhacumbe et al. 2019; Bartley et al. 2021; Fávero et al. 2020). In clinical practice, there is no definitive evidence of resistance; nonetheless, reduced drug efficacy (Vlaminck et al. 2019; Walker et al. 2021) is a major concern (Zeleke et al. 2020). Studies conducted using nucleic acids derived from a variety of sources, including adult worms, dissected or concentrated eggs, or stool samples, to detect SNPs in human STHs, are indicated in Table 3. The presence of the common resistance markers in human STHs further provides compelling evidence for how patterns of resistance in veterinary parasites can emerge in related human parasites (O'Halloran 2021). What is currently uncertain is whether any of these SNPs are influencing BZ efficacy against human STHs (Furtado et al. 2019; Grau-Pujol et al. 2022; Matamoros et al. 2019; Orr et al. 2019; Zuccherato et al. 2018). However, Diawara et al. (2013) concluded that ALB exerts selection pressure on the β-tubulin gene at position 200 in *T. trichiura*, which could explain the moderate ALB efficacy against human trichuriasis.

**Table 3:** Review of referenced studies for the detection of putative resistance SNPs in human STHs

Codon position	Technique	STH spp.	Frequency of SNP occurrence	Country	References
1. F200Y	ARMS-PCR	*A. lumbricoides*	Low	Brazil	(Furtado et al. 2019)
2. E198A	SmartAmp2	*N. americanus*	x	–	(Rashwan et al. 2016)
3. E198A & F200Y	Nested PCR and PCR-RFLP	*N. americanus*	Low	Brazil	(Zuccherato et al. 2018)
4. F167Y & E198A		*A. lumbricoides*	x	Brazil	(Zuccherato et al. 2018)
5. F167Y, F200Y, E198A	PCR and sequencing	*N. americanus*	Low	Ghana	(Orr et al. 2019)
6. F167Y, F200Y, E198A	PCR and pyrosequencing	*T. trichiura*, *N. americanus*	x	Mozambique	(Grau-Pujol et al. 2022)
7. F167Y, E198A, E198L, F200Y	Deep amplicon sequencing	*A. lumbricoides*, *A. suum*	x	Ethiopia, Tanzania, Belgium	(Roose et al. 2021)
8. F167Y, F200Y, E198A	Pyrosequencing	*T. trichiura*, *N. americanus*	x Low	Mozambique	(Gandasegui et al. 2021)
9. F167Y, F200Y, E198A	Semi-nested PCR, sequencing	*A. lumbricoides*, *T. trichiura*	x	Honduras	(Matamoros et al. 2019)

x means SNP was not detected

Given the current increase in pharmacological pressure by MDA projects, there is a reason to be concerned that AR may spread to the human species. This imminent threat needs ongoing research to understand the mechanisms of resistance and develop innovative techniques to tackle the fundamental issue of helminthiasis that minimize the usage of chemical control.

## Drivers of resistance

According to Kebede (2019), it is critical to distinguish between decreased efficacy and AR; however, (1) doing so, in reality, is difficult, (2) many potential confounding factors may affect an AH’s efficacy and should be eliminated before assuming AR, and (3) the confounding factors have been extensively investigated in veterinary intestinal nematode infections, but less so among species infecting humans. Fissiha and Kinde (2021) added that AR development is a highly multifaceted process that is affected by the host, the parasite, the type of AH and its usage pattern, animal management, and climatic characteristics. Therefore, to limit its effects or slow its spread while effectively controlling helminthiasis, a thorough understanding of the conditions that predispose to AR is critical.

### Frequency of drug administration

The frequency of treatment plays a significant role in the development of drug resistance, especially if the same group of drugs is frequently administered. A higher drug pressure results in a faster selection of resistant helminth strains (Fissiha and Kinde 2021; Kaplan 2020). The fundamental principle for selection is that regular treatment offers the surviving parasites a reproductive and replication advantage over the susceptible parasites. High treatment frequency was found to be responsible for thiabendazole (TBZ) and ALB resistance recorded in sheep in Limpopo Province, South Africa (Mphahlele et al. 2021). As a result, the question of whether this is likely to occur in human STHs arises. This is especially significant given that the rapidly expanding MDA programs for human STHs rely almost entirely on BZs for treatment (WHO 2023a).

### The use of anthelmintics in sub-optimal doses

Improper weighing to estimate the dose rate of an anthelmintic in particular and a drug in general in veterinary medicine can result in underdosing, allowing heterozygous resistant worms to survive and therefore contribute to the selection of resistance strains (Fissiha and Kinde 2021). One study, in particular, noted that none of the sheep flocks under study had adequate weight estimation for calculating the correct AH dose but relied solely on the visual appraisal of animals to determine their weight, which represented a high risk for underdosing (Mphahlele et al. 2019b). In the findings of Mphahlele et al. (2019b), drenching practices, commonly practiced in resource-poor farmers, may have had an immense contribution to the development of AR in the Limpopo farm.

Human therapeutic regimens are frequently sub-optimal: AHs are administered in single doses and do not always achieve 100% efficiency, an approach frequently utilized in public health helminth control programs to lower the expenses of treatment campaigns in developing countries (Moser et al. 2019; Partridge et al. 2020). As a result, the small percentage of resistant parasites released into the environment contaminate soil and pasture, leading to the development of the bulk of resistant generations and, ultimately, the spread of AR.

### Other factors

Some other factors that can also contribute towards the development of AR include introducing resistant parasites through animals transported from country to country. Furthermore, factors like the number of genes involved in resistance and their dominance or recessiveness, frequency of resistance alleles in the initial untreated population, and biological fitness of unselected worms may also contribute to the development of resistance (Kotze et al. 2020).

## The concept of One-Health

**Fig. 1:**
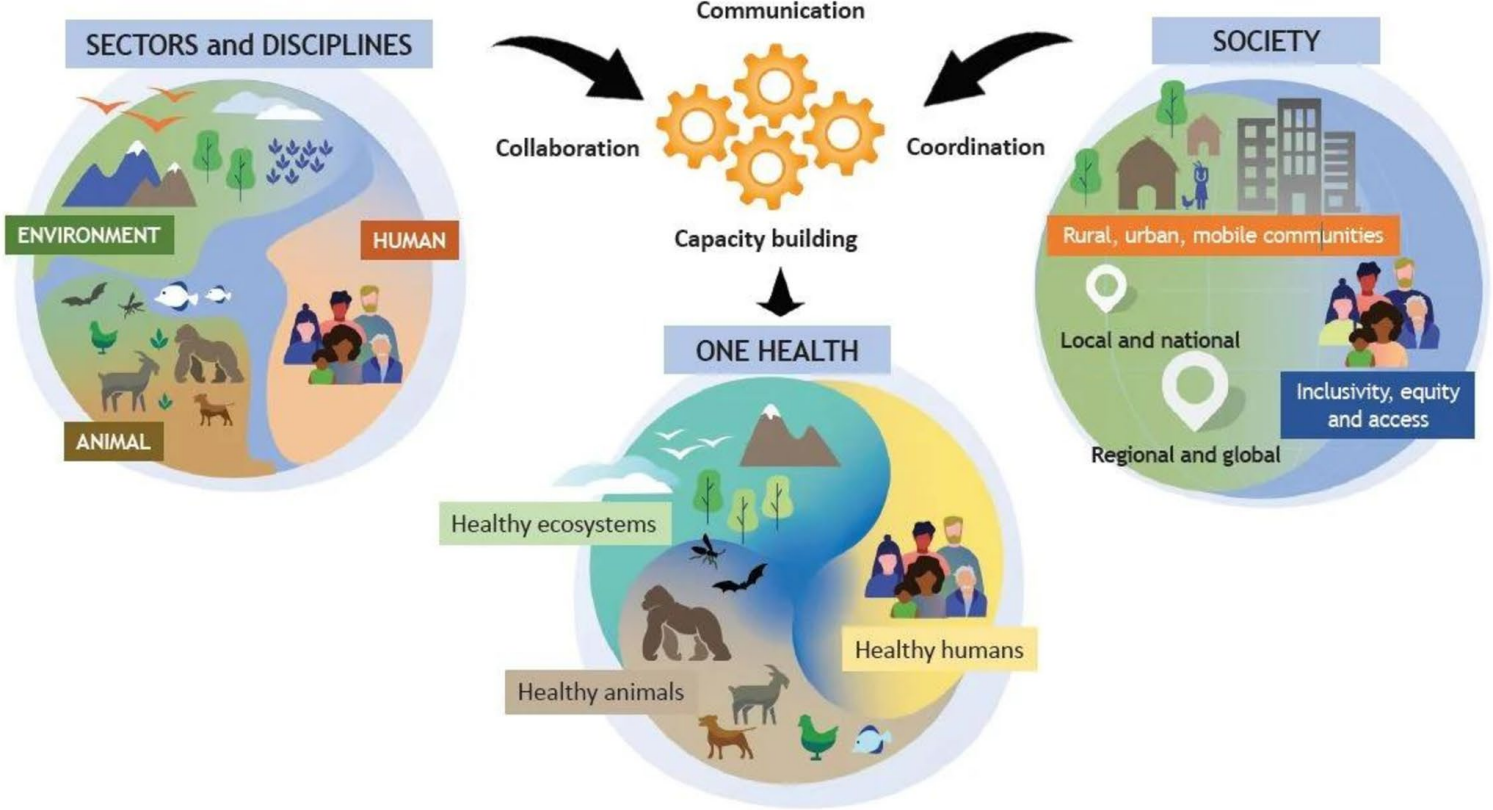
Pictorial definition of One-Health by OHHLEP (2021)

The One-Health approach or concept is a worldwide strategy that refers to a collaborative and interdisciplinary approach at local, national, and global levels to improve the health of people, animals, and the environment (OHHLEP 2021; Riley et al. 2021). In recent years, One-Health has gained appeal as a strategy for dealing with emerging infectious illnesses of public health relevance, including zoonoses, supported by the WHO, the Food and Agriculture Organization of the United Nations (FAO), and the World Organization for Animal Health (OIE) (WHO 2017). Figure 1 depicts the One-Health High-Level Expert Panel (OHHLEP)’s integrated and unifying definition of One-Health (OHHLEP 2021). This derives from the knowledge that animal health and the environment in which they live are inextricably interconnected (OHHLEP 2021), and also that the majority of human diseases originate from animal sources (CDC 2022; Overgaauw et al. 2020). Furthermore, drivers such as changes in climate and land use, unsustainable agricultural practices, globalization, and the wildlife trade provide multiple opportunities for pathogens to evolve into new forms, making spillover events from animals to humans more frequent and intense (OIE 2023). With this in mind, mobilizing experts from human health, animal health, environmentalists, and other areas of expertise, health threats can be better monitored and controlled through enhanced coordination, collaboration, and communication, with the ultimate goal of achieving optimal health outcomes for all (CDC 2022).

STHs are examples of diseases that must be addressed through One-Health efforts. This is because STHs mainly affect populations in places with sanitation challenges as STH transmission particularly occurs through contact with soil contaminated with helminth eggs or larvae (WHO 2023a). Contributing factors of animal, environmental, and human origin may affect multiple aspects associated with STH infections. For instance, vertebrate animals such as dogs living near humans in regions lacking veterinary services significantly influence the risk of zoonotic canine hookworm transmission (Colella et al. 2021; Massetti et al. 2020; Traub et al. 2021; Zendejas-Heredia et al. 2021), *T. trichiura* (Areekul et al. 2010), *A. duodenale* (Fetouh 2003), and *N. americanus* (Boyko et al. 2020). Table 4 lists zoonotic STHs, their risk of transmission, and their impact on human health. The lack of adequate sanitary and hygiene facilities exacerbates the contamination of soil, food, and clean water with infective eggs/larvae, increasing infection risks. When infected hosts defecate on soils (beaches, backyards, children’s playgrounds), the spread of infective parasite stages and the risk of infection increases (Delaluna et al. 2020; Ngcamphalala et al. 2020; Traub et al. 2021). Human factors such as age may influence the pathogenesis of disease caused by STH infection (high *Ascaris*, *Trichuris*, and *Toxocara* worm loads lead to more severe illnesses in children) (Else et al. 2020). Aside from coevolution, greater human occupancy in animal habitats and close contact or affiliation with pets and domestic animals in human dwellings, augmented by individual variables and behaviors, allow for zoonotic pathogen exposure (Kajero et al. 2022; WHO 2020). Pet ownership is increasing in many households, and too close contact or association can be harmful to pet owners (Kamani et al. 2021), particularly in areas where the owners are unaware of the associated zoonotic disease risks and thus do not take the necessary precautions to avoid any potential health hazards (Moro and Abah 2019).

**Table 4:** Zoonotic STHs, risk of transmission, and impact on human health

Disease	Parasite	Host	Risk for transmission	Health impact	References
Ancylostomiasis	*A. caninum*, *A. ceylanicum*, *U. stenocephala*, *A. braziliensis*, *A. duodenale*, *N. americanus*	Dogs, humans, foxes, cats, non-human primates, wild dogs, pigs	Skin penetration by filariform larvae in soil/vegetation/oral ingestion of infective larvae	Cutaneous larva migrans, acute and chronic anemia, eosinophilic enteritis, diarrhea, ileitis	(Fetouh 2003; Massetti et al. 2020; Stracke et al. 2020; Traub et al. 2021; Colella et al. 2021; Boyko et al. 2020
Ascariasis	*A. suum*, *A. lumbricoides*, *Baylisascaris procyonis*	Pigs, humans, dogs, raccoons	Ingestion of embryonated eggs in water, food, hands, fur	Abdominal pains, intestinal obstruction and potential perforation, appendicitis, or nasopharyngeal expulsion, malnutrition, growth retardation, impaired cognitive development, neurological disease, death	(CDC 2019a; Sadaow et al. 2018)
Trichuriasis	*T. suis*, *T. vulpis*, *T. trichiura*	Dogs, humans, pigs	Ingestion of embryonated eggs in soil-contaminated hands, water, food	Rectal prolapse; frequent, painful passage of stool that contains a mixture of mucus, water, and blood; severe anemia; growth retardation; and impaired cognitive development	(Areekul et al. 2010; Jacob and Lorber 2016)
Toxocariasis	*Toxocara canis*	Pet dogs, wild canids	Fully embryonated eggs/larvae in raw meat of paratenic hosts, e.g., chicken	*T. canis* infections lead to visceral larva migrans, ocular and neuro-toxocariasis in humans	(Massetti et al. 2022)

With groundwater levels dwindling, wastewater treatment and utilization have become critical in small- and large-scale agricultural areas. However, the extremely high levels of STH eggs found in wastewater and sludge from developing countries reviewed by Amoah et al. (2018) far exceed limits set in the WHO guideline for wastewater/sludge reuse (≤ 1 helminth egg per gram or liter of sludge or wastewater) intended for unrestricted agriculture (WHO 2006), endangering the health of communities in contact with the wastewater/sludge via various exposure routes (Amoah et al. 2018). Scientific evidence shows the presence of traces of AHs in plants, waterways, and soils (Li et al. 2020; Mesa et al. 2020; Mooney et al. 2021; Navrátilová et al. 2021; Porto et al. 2021), and even promote drug resistance development in sheep GINs (Dimunová et al. 2022). As a result, understanding the spread of resistance in the environment and its influence on human health is critical.

## One-Health approach to anthelmintic drug resistance

The One-Health approach ensures that health concerns are addressed in an integrated and holistic manner, offering a more comprehensive awareness of the issues from a human-animal-environment standpoint and presenting potential solutions that would be unattainable if the issues were to be tackled individually. AR is linked to humans, animals, and the environment because AHs have been excessively utilized in many fields of agriculture, livestock husbandry, and human medicine, contaminating plants, fresh waterways, and soils, potentially promoting the development of AR in humans and animals following long periods of exposure. As therapeutics and prophylactics, AHs are important in human and animal health. However, with the current escalated AR in animal husbandry and the emergence in clinical medicine, the future of STH control is unknown. Thus, to protect current and future AHs, a shift regarding how AHs are used ought to be executed. To incorporate One-Health concepts into addressing the problem of AR, stakeholders from various sectors such as human and animal health, agriculture, and environmental conservation need to come together to develop and implement integrated approaches that address the root causes of AR. This section goes into detail about some ways.

### Implementing targeted deworming protocols

The AR problem in livestock has had significant financial consequences. While this is horrendous by itself, widespread AR in humans, if it occurs, would become a “serious public health problem” with increased illness and mortality among at-risk groups (Tinkler 2020). Implementing measures such as selective drug use only when required (to treat severely sick individuals) aims to slow the pace of resistance development, hence extending the lifetime of currently effective AHs. For example, where *Haemonchus contortus*, the major killing parasite of sheep and goats, is the predominant GIN, the FAMACHA program allows individual animal assessment to determine the development or severity of anemia in the flock or herd (Cintra et al. 2019; Prashanth et al. 2020; Senoamadi et al. 2022), thereby deworming only the animals showing symptoms of heavy parasite infection, rather than the “blanket” or mass treatment. Such targeted selective anthelmintic treatment (TST) procedures not only reduce the use of AHs and the number of animals dewormed but also chemical residues in meat animals and any associated environmental health problems (Höglund et al. 2013). By limiting the use of AHs in this way, the selective pressure on parasites to evolve resistance is reduced. Additionally, targeted deworming of only sick/infected individuals/populations slows the spread of AR by limiting the likelihood of resistant parasites spreading to uninfected populations.

Despite the possibility of generating resistant helminths, repeated mass treatment programs, known as PCs, that typically employ a single class of medications, BZs, administered to children attending school, currently expanded to community-wide level, remain the cornerstone for combating human STHs (Pilotte et al. 2022; Tinkler 2020; WHO 2023a). This was demonstrated to contribute more than a 50% reduction in the amount of DALYs lost each year (WHO 2023a), with others forecasting as much as 75% of all morbidity averted, if current treatment targets (< 2% morbidity) are met (Walson 2021; WHO 2019a). However, previous research has indicated that just treating schoolchildren is unlikely to result in transmission cessation because the untreated adult population would continue to contribute to the reservoir of infectious agents, sustaining the cycle of reinfection (Pilotte et al. 2022). As a result, increasing focus has been placed on implementing selective treatment of all infected persons to attain appropriate prevalence thresholds below which helminth parasite populations cannot continue transmission, resulting in transmission termination (Werkman et al. 2018a, 2018b). Additional measures, such as a reduction in poverty (Walson 2021) and significant improvements in access to clean water, sanitation, and hygiene (WASH) (Pilotte et al. 2022; WHO 2023a; Zeng et al. 2019), will reduce reinfections and the need for frequent deworming.

### Incorporating alternative control methods

The rising prevalence of AR especially in animal husbandry has raised questions regarding the long-term viability of the existing chemotherapeutic strategy in treating STHs in humans and animals. In this regard, the search for alternative/novel parasite management approaches to reduce parasite transmission and dependency on AHs is deemed critical in curbing the spread of AR. Thus, the goal is to keep host-parasite contact to a minimum such that helminths have a minimal influence on host well-being and/or performance. Rotating 2-month-old weaning sheep and goats through safe pastures ahead of the adults, for example, would reduce their exposure to high numbers of infective larvae (Zekarias and Toka 2019). Keeping animals under a zero grazing system restricts their access to any vegetation, thus lowering the risk of parasitism and preventing reinfection. As parasite species differ between host species, multi-species grazing and/or alternate grazing of cattle or horses with sheep or goats offers important benefits to parasitic nematode control (Bambou et al. 2021; Verma et al. 2018). Provision of clean water free from fecal matter, proper drainage in the animal shed, avoiding overstocking in pastures and pens, and isolation of newly acquired animals to be aggressively dewormed to prevent the introduction of drug-resistant worms are some management practices for sustainable parasite control. Using host genetics to produce parasite-resistant and robust breeds like Red Maasai (Benavides et al. 2015) and the Brazilian Morada Nova (Marei et al. 2020) sheep is expected to be a long-term defense against internal parasites, such as the hematophagous *H. contortus* spp. Aside from targeting parasitic stages within the animal, biological control targeting free-living infective larvae on pasture by use of certain micro-fungi, particularly *Duddingtonia flagrans*, dung beetles, and earthworms, aims to reduce the number of infective stages available to susceptible grazing livestock (Canhão-Dias et al. 2020; Mendoza-de Gives et al. 2018; Szewc et al. 2021). Vaccination has been proposed as a promising alternative control method for STHs. Although no vaccines are currently available for human STHs, vaccines, such as Barbervax (Nisbet et al. 2016), were developed for *H. contortus*, a voracious blood-sucking nematode of small ruminants causing high morbidity and hitting hard the economy of sheep farming worldwide (Singh et al. 2019). Overall, these alternative control methods help promote the principles of the One-Health approach by minimizing incidences of parasite infections in humans and animals, while reducing parasite stages in the environment, thus improving and protecting the health and well-being of the entire communities. Additionally, these methods are more sustainable and have a lower environmental impact than the traditional chemical approach, thus protecting the environment.

### Developing and promoting the use of effective diagnostic tools

Diagnostic tools are essential in identifying nematode parasite infections as well as determining drug resistance levels. Technology advancements such as Smartphone applications (Saeed and Jabbar 2018) and FECPAK^G2^ (Rashid et al. 2018) have enabled on-farm sample processing and parasite detection. In this case, large numbers of samples can be prepared and then later analyzed. Further digital images remain available for referencing and auditing purposes. On the other hand, molecular techniques such as qPCR exhibit high sensitivity and can differentiate human and animal species based on their DNA/RNA sequences (Dos Santos et al. 2022; Furtado et al. 2020; Ngcamphalala et al. 2020; Palma et al. 2019; Xu et al. 2021; Zhou et al. 2021), significantly improving diagnostic sensitivity and offering the prospect of diagnosing even very low-level infection intensities (Benjamin-Chung et al. 2020; Dunn et al. 2020). This enables better monitoring of parasite infections and their response to treatment. Additionally, the advent of next-generation sequencing (NGS) technologies has revolutionized the use of molecular techniques for understanding complex microbial communities, in fecal (Kirstahler et al. 2022) and a variety of other sample matrices including environmental samples such as wastewater (Azli et al. 2022; Brumfield et al. 2022; Garner et al. 2021; Grundy et al. 2023; Li 2019).

Detecting the presence of AR is crucial in reducing its severity (Kotze et al. 2020). Phenotypic techniques, such as the larval development test (LDT) and the fecal egg count reduction test (FECRT), are extensively employed to detect AR in livestock GIN parasites (Bosco et al. 2020; Kelleher et al. 2020; Mphahlele et al. 2021; Potârniche et al. 2021) and for assessment of drug efficacy in terms of egg reduction rate (ERR) for human STH (Olliaro et al. 2022; Subba and Singh 2020). Similarly, genotypic approaches based on PCR have been significantly refined for the detection of BZ resistance (Nath et al. 2022; Roose et al. 2021; Sargison et al. 2019), and recently levamisole (Araújo-Filho et al. 2021), by amplifying the specific genetic mutations associated with resistance. As a result, targeted treatment/interventions can be implemented. Additionally, molecular tools can be used to monitor the spread of resistant helminth strains. This allows for current and informed decisions regarding drug usage, as well as the development of effective control measures. However, each technique has benefits and drawbacks in terms of accuracy (Sangster et al. 2018), reproducibility, cost, and time spent (Kotze et al. 2020). Overall, the use of molecular techniques in the fight against anthelmintic resistance is a promising approach that can help preserve the efficacy of these important drugs and protect human and animal health.

Nonetheless, the detection of AR in animals and humans has received more attention than the environmental aspect of AR. Water-based epidemiology (WBE) approaches, for example, can provide an overview of a community’s overall health by monitoring the presence of biological or chemical indicators dissipated in a pooled sample of sewage/sludge or wastewater (O’Keeffe 2021). Environmental sampling can inform and improve prevention, intervention, and control (Larsson and Flach 2022; O’Keeffe 2021; Prieto Riquelme et al. 2022; Sims and Kasprzyk-Hordern 2020), before spillover into humans, especially in this era of rapid population growth and environmental changes. However, the absence of data on the existence and quantities of AR genes in environmental samples makes it impossible to establish their impact on human health. Similarly, further research is needed to detect the existence and quantities of AR genes in environmental samples, particularly in light of the increasing incidence of AR in livestock and the high possibility of its emergence in human STHS.

### Encouraging responsible use of AHs among farmers, veterinarians, and stakeholders

Due to many years of unethical and unregulated use, anthelmintic drug resistance threatens to collapse animal agribusiness. Responsible and prudent use of AHs is one way to slow down resistance. This calls for a concerted effort by all stakeholders involved in handling medicines containing anthelmintic compounds.

Education plays a crucial role in promoting the responsible use of veterinary drugs and preventing drug resistance. Proper understanding and training on the appropriate use of veterinary drugs can enhance their effectiveness in treating animal illnesses while minimizing the development of drug resistance. Regular training creates awareness among veterinarians, animal owners, and other stakeholders who become familiar with current scientific thinking on the correct choice, use/administration of anthelmintic chemicals, as well as current issues regarding AR and its diagnostic methods. While engaging with farmers regarding animal health plans, competent veterinarians will provide sound animal husbandry methods such as good sanitation and hygiene practices which help minimize incidences of animal illnesses, as well as non-chemical parasite control methods, thus diminishing the need for anthelmintic chemicals. Additionally, animal owners and veterinarians should be informed of the potential impact of AR on animal health and the potential risks to human health. This can help to motivate stakeholders to adopt responsible drug use practices and to take steps to prevent the development of resistance. The veterinarians have a role on the front line in detecting and reporting safety and efficacy issues to the competent authority and testing for and providing localized accounts of AR. Possible training programs should be readily available to individual large- and small-scale food animal producers as they have a direct role in the responsible and prudent use of anthelmintic chemicals in their animals, hence promoting animal health and food safety. Post-training monitoring is important, especially to evaluate the correct use of non-chemical farming approaches (Maia et al. 2015).

National and municipal governments may adopt international-compliant laws and guidelines for the use and distribution of AH medicines. This necessitates the implementation of proper regulatory controls and procedures at the national, international, or regional levels, as well as coordination by the respective authorities, to prevent the unauthorized manufacture, importation, distribution, storage, and use of unlicensed, substandard, or fraudulent AH products (British Veterinary Association 2023; European Medicines Agency 2021; Vinny and Hayley 2019). Clear guidelines on the dosages, administration methods, and withdrawal periods for different animal species, and manufacture and expiry dates should be indicated on medicine containers. Furthermore, by regular inspections and penalties for noncompliance, governments can monitor and enforce compliance with these regulations and recommendations. Governments may help to ensure that AH medicines are used ethically and sustainably, lowering the possibility of drug resistance and enhancing the health and welfare of the environment, animals, and humans, as well as ensuring the safety of the food chain, through the implementation of these measures.

### Conducting ongoing anthelmintic resistance monitoring and surveillance

Monitoring and surveillance are critical components in the management and control of AR. These activities help to detect diminishing drug efficacy due to the development of AR in specific hosts and parasites, as well as track trends in AR, hence providing a global pattern and informing future measures to combat the problem. Monitoring involves the regular collection and analysis of data on anthelmintic efficacy, while surveillance involves targeted data collection to identify emerging AR trends and risk factors. One of the essential tools for monitoring and surveillance of AR is the FECRT, which measures the efficacy of anthelmintics in reducing the number of parasite eggs in fecal samples. High levels of egg reduction indicate high efficacy, while low levels indicate reduced efficacy and the potential development of resistance. Other tools for monitoring and surveillance include molecular techniques that detect genetic markers associated with AR, such as mutations in the beta-tubulin gene associated with BZ resistance. Monitoring is concentrated in veterinary settings (Avramenko et al. 2020; Dauparaitė et al. 2021; Hinney et al. 2020; Queiroz et al. 2020), and only one project, the Starworms (STop Anthelmintic Resistant WORMS), has been piloted for human STHs, to measure drug efficacy and further investigate the presence and distribution of AR-related SNPs (Vlaminck et al. 2020). Thus, the efficacy of clinical antihelminthics and the presence of AR in human STH species are currently unknown.

Environmental contamination with anthelmintic residues is a One-Health concern. AHs are often excreted into the environment in relatively high amounts (Mooney et al. 2021), with parent drugs or their transformation products (TPs) detected in various environmental settings (Li et al. 2020; Mesa et al. 2020; Mooney et al. 2021; Navrátilová et al. 2021; Porto et al. 2021), yet insufficient monitoring and surveillance limits information available on the environmental occurrence of AR genes, and the possible spillover to exposed humans and animals. Thus, the true scale and potential burden of AR may be underestimated. This is important, considering that wastewater may contain sewage from livestock farms and wet markets, active hotspots for amplification of infectious agents, including zoonotic pathogens (Xiao and Zhang 2023), antimicrobial-resistant (AMR) organisms, and genes.

By regularly monitoring and surveying for AR, public health officials can identify areas with high levels of resistance and target interventions, such as the use of alternative anthelmintics or non-chemical control measures. This approach can help reduce the spread of resistance and preserve the efficacy of existing AHs, as well as prevent environmental contamination. In addition, monitoring and surveillance can inform future measures to combat AR by identifying emerging trends in resistance and risk factors, enabling researchers to develop new AHs with different modes of action, implement targeted breeding programs, or develop alternative control measures such as vaccines or biological control agents.

## Conclusion and recommendations

In conclusion, the use of AHs in control of STHs and other GINs remains the cornerstone approach, and hence, it is crucial to use the current effective AHs carefully to minimize the impact of AR. It is recommended that One-Health strategies, such as targeted deworming, precise diagnosis of specific parasites and level of resistance, expanding the monitoring and surveillance of AR to environmental matrices such as wastewater, raising awareness through education, and employing alternative control methods, be adopted to reduce overreliance on anthelmintic chemicals while also sustainably reduce parasite burdens across humans, animals, and the environment, thereby slowing down the development of AR. The monitoring and surveillance of AR through drug efficacy trials in clinical settings must be intensified to establish the link between AR-related SNPs, response to AH treatment, and AR. Strict measures ensuring the ethical and sustainable use of AHs are implemented by governments to ensure the safety of food and promote the health of humans, animals, and the environment. More research should look into understanding the spread of resistance in the environment and its influence on human and animal health.

## Data Availability

Not applicable.

## Abbreviations

*AHs*: anthelmintics
*AR*: anthelmintic resistance
*FAMACHA*: FAffa MAlan CHArt
*GABA*: 4-aminobutyric acid
*MPTL-1*: a subtype of nicotinic acetylcholine receptors
*nAChR*: nicotinic acetylcholine receptor
*Pgps*: P-glycoproteins
*STHs*: soil-transmitted helminths
